# A Comparative Study of the Antioxidative Effects of *Helichrysum italicum* and *Helichrysum arenarium* Infusions

**DOI:** 10.3390/antiox10030380

**Published:** 2021-03-03

**Authors:** Katja Kramberger, Zala Jenko Pražnikar, Alenka Baruca Arbeiter, Ana Petelin, Dunja Bandelj, Saša Kenig

**Affiliations:** 1 Faculty of Health Sciences, University of Primorska, 6310 Izola, Slovenia; katja.kramberger@fvz.upr.si (K.K.); zala.praznikar@upr.si (Z.J.P.); ana.petelin@fvz.upr.si (A.P.); 2 Faculty of Mathematics, Natural Sciences and Information Technologies, University of Primorska, 6000 Koper, Slovenia; alenka.arbeiter@upr.si (A.B.A.); dunja.bandelj@upr.si (D.B.)

**Keywords:** *Helichrysum*, medicinal plants, infusions, phenolic compounds, antioxidative potential

## Abstract

*Helichrysum arenarium* (L.) Moench (abbrev. as HA) has a long tradition in European ethnomedicine and its inflorescences are approved as a herbal medicinal product. In the Mediterranean part of Europe, *Helichrysum italicum* (Roth) G. Don (abbrev. as HI) is more common. Since infusions from both plants are traditionally used, we aimed to compare their antioxidative potential using in vitro assays. Two morphologically distinct HI plants, HIa and HIb, were compared to a commercially available HA product. Genetic analysis using microsatellites confirmed a clear differentiation between HI and HA and suggested that HIb was a hybrid resulting from spontaneous hybridization from unknown HI subspecies. High-performance liquid chromatography–mass spectrometry analysis showed the highest amounts of hydroxycinnamic acids and total arzanol derivatives in HIa, whereas HIb was richest in monohydroxybenzoic acids, caffeic acids, and coumarins, and HA contained the highest amounts of flavonoids, especially flavanones. HIa exhibited the highest radical scavenging activity; it was more efficient in protecting different cell lines from induced oxidative stress and in inducing oxidative stress-related genes superoxide dismutase 1, catalase, and glutathione reductase 1. The antioxidative potential of HI was not only dependent on the morphological type of the plant but also on the harvest date, revealing important information for obtaining the best possible product. Considering the superior properties of HI compared to HA, the evaluation of HI as a medicinal plant could be recommended.

## 1. Introduction

*Helichrysum arenarium* (L.) Moench (hereinafter abbreviated as HA) has a long tradition in European ethnomedicine as a medicinal plant with many known activities; the EU monograph supports its use for digestive disorders, feeling of fullness and bloating, and, importantly, no side effects are reported [1]. An assessment report of the European Medicines Agency (EMA) Committee on Herbal Medicinal Products (HMPC) supports its stimulatory role in bile flow and in the release of cholesterol into the bile. HA has also been used in chronic liver inflammatory disease and as a scavenger of free radicals [2]. HA inflorescences are the only herbal medicinal product from the *Helichrysum* genus (*Asteraceae* family) approved by the HMPC and currently available on the market as such.

While HA can be found mainly in Poland, Russia, Lithuania, and Latvia [3], in the Mediterranean region of Slovenia and in other regions with similar climatic conditions, such as coastal Croatia, Italy, Spain, Portugal, and Corsica, *Helichrysum italicum* (Roth) G. Don (abbreviated as HI) is more common, both as a wild and as a cultivated plant [4]. Similar to HA, HI is widely used in traditional medicine but has not yet been recognized and evaluated by the HMPC. Moreover, scientific data are mostly limited either to topical applications in wound healing or to the investigation of individual compounds in in vitro models [5,6]. The antimicrobial activity of ethanol, methanol, and acetone extracts and essential oil is also well documented [7,8,9]. 

Lately, much attention has been paid to natural antioxidants and their ability to delay the progression of chronic diseases related to the formation and the activity of reactive oxygen species (ROS) and other free radicals [10,11]. Among these natural products, various herbal extracts containing numerous plant secondary metabolites exhibit the ability to scavenge ROS. Herbs are very often used to prepare recreational tea and are thereby important sources of antioxidants in different cultures [12]. Although the English term “tea” refers to the infusion made from the leaves of a tea plant (*Camellia sinensis* (L.) Kuntze), in colloquial language it also refers to the wide variety of locally grown herbs used in different regions of the world for recreational tea. Among the most important ones in Europe are Roman chamomile (*Chamaemelum nobile* (L.) All.), red raspberry (*Rubus ideus* L.), and lemon balm (*Melissa officinalis* L.) whereas in southern Europe, chamomile (*Matricaria chamomilla* L.) and lemon thyme (*Thymus pulegioides* L.) are frequently used [12]. Due to the intense fragrance of HI, its plantations are primarily dedicated for use in the cosmetic industry. However, in traditional medicine, HI is also used in the diet, mainly for the preparation of infusions to treat respiratory and digestive conditions [9]. Recently, it was confirmed by Kramberger, et al [13] that hot water extracts prepared from dried HI aerial parts contain high amounts of bioactive compounds and have comparable antioxidative activity to the ethanol and methanol extracts.

Several subspecies (ssp.) of HI are known, but genetic or morphological description of the plant material used is often lacking in studies [9]. While it has been shown that the method of extract preparation crucially contributes to the heterogeneity of extracts, genetic background is also a major factor influencing the profile of the different bioactive compounds [13]. This suggests that comprehensive plant characterization would be important to obtain the best health beneficial effects of infusions or a recreational tea. 

Therefore, we analyzed the content of the main bioactive compounds and the antioxidative properties, tested with cellular and non-cellular in vitro methods, of infusions prepared from two morphologically distinct HI plants (HIa and HIb) in comparison to the commercially available HA. In addition to evaluating HI and comparing it with a recognized medicinal plant, we also aimed to provide information relevant for the farmers to select appropriate plant material for their plantations to obtain the most advantageous health beneficial effects of their crop.

## 2. Materials and Methods

### 2.1. Plant Material

The analyzed plant material included two *Helichrysum* species: dried flower heads of commercially available *Helichrysum arenarium* (L.) Moench tea (sample HA), purchased from Flora Ltd., Rogatec, Slovenia, and *Helichrysum italicum* (Roth) G. Don plants (samples HIa and HIb) cultivated in two Slovenian plantations. Within the *H*. *italicum* group, two morphological types were identified according to their morphological differences: plant *H*. *italicum* a (sample HIa, characteristic of common plants) and plant *H*. *italicum* b (sample HIb, characteristic of one sample). Based on taxonomically relevant characters [14], morphological type HIb differs from type HIa, mainly by a shorter leaf length, a clear presence of axillary leaf fascicles, a leaf margin undulation, and a lower number of capitula per synflorescence. The HIa plants originated from a commercial, private plantation in Dragonja (45°27′05″ N 13°41′31″ E), Slovenia. The HIb plants were collected from the ex situ experimental collection of the University of Primorska, established in 2018 near Ankaran (45°34′19″ N 13°46′32″ E), Slovenia. Plants from both plantations were grown under similar ecological conditions of a sub-Mediterranean climate. Herbarium specimens of both HIa and HIb plants were deposited at the University of Primorska, Faculty of Mathematics, Natural Sciences and Information Technologies, Slovenia, under accession numbers HIa_UP20 and HIb_UP20.

### 2.2. DNA Extraction and Microsatellite Analysis

Total genomic DNA was extracted from fresh leaves of cultivated HIa HIb and from dried flower heads of HA using the modified cetyltrimethyl ammonium bromide-polyvinylpyrrolidone (CTAB-PVP) protocol [15]. DNA extracts from plants from both collection fields and of commercial tea Flora Ltd. were deposited in the genetic laboratory of the University of Primorska, Slovenia under the accession numbers HIaUP_1-30 (*H. italicum* plant a), HIbUP_1 (*H. italicum* plant b), and HAUP_1-7 (*H. arenarium*).

The concentration of DNA was determined by Qubit^TM 3^ (Thermo Fisher Scientific, Waltham, MA, USA). A set of eight newly developed microsatellite primers (HiUP-01, HiUP-02, HiUP-09, HiUP-13, HiUP-16, HiUP-18, HiUP-22, HiUP-24) designed by Baruca Arbeiter, et al. [15] were selected for genetic analysis. Amplification was carried out using a DNA Engine Thermal Cycler 200 (Bio-Rad Laboratories, Hercules, CA, USA) containing 1x AllTaq PCR Buffer, 2 mM MgCl_2_, dNTPs (0.2 mM of each dNTP), 1x Q-Solution, 1.25 U of AllTaq DNA polymerase (Qiagen, Hilden, Germany), 0.2 µM of each locus-specific primer (synthesized by IDT), 0.25 µM of universal primer M13(-21) labelled with 6-FAM, VIC, PET or NED (Applied Biosystems, Foster City, CA, USA), and 40 ng of template DNA. The two-step amplification profile consisted of an initial denaturation at 94 °C for 5 min, followed by 30 cycles (step 1) of 94 °C for 30 s, 56 °C for 45 s and 72 °C for 45 s, followed by eight cycles (step 2) of 94 °C for 30 s, 53 °C for 45 s and 72 °C for 45 s. Final extension was carried out at 72 °C for 8 min [16]. Fragment analysis was conducted using SeqStudio^TM^ Genetic Analyzer (Applied Biosystems, Foster City, CA, USA), and the allele calling was done with GeneMapper version 4.1 (Applied Biosystems, Foster City, CA, USA).

### 2.3. Infusion Preparation

For chemical analysis, HIa and HIb aerial parts were harvested from the 24th to 29th of June 2019 and air-dried. For analysis of the optimal harvest period, the material was harvested in May and June 2020. Plant material (HIa, HIb and HA flower heads) was milled to 2–3 mm fine particles and stored at room temperature in a dry and dark place until further use. For the analysis of separate plant parts, samples of HIa were separated to green parts (stems and leaves) and flowers. Fresh infusions were prepared from the same stock material for all cell culture experiments and for chemical analysis by infusing 0.5 g of dried plant material in 100 mL of boiling water. After a 10-min incubation, infusions were filtered through Whatman paper and passed through a 0.2 µm cellulose acetate membrane filter (Macherey-Nagel GmbH & Co KG, Düren, Germany). For the chemical analysis, samples were screened in the same dilution.

### 2.4. Chemical Analysis

High-performance liquid chromatography–mass spectrometry (HPLC-MS) analysis was performed using an Agilent 1260 Infinity II HPLC system (Agilent Technologies, Santa Clara, CA, USA) equipped with a diode array detector (DAD, model G7115A) and coupled to an Agilent 6530 Accurate-Mass Quadrupole Time-of-Flight (Q-TOF) MS system equipped with an Agilent Jet Stream dual electrospray ionization (ESI) source. The HPLC system included a binary pump (model G7112B), Agilent 1260 Autosampler (model G7129A) and a Poroshell 120, EC-C18, 2.1 × 150 mm, 2.7 µm column (693775-902, Agilent Technologies, Santa Clara, CA, USA). Separation was obtained with a linear gradient of (A) water + 0.1% formic acid (*v*/*v*) and (B) acetonitrile/methanol (50:50, *v*/*v*), starting at 3.0% B and increased to 100.0% B in 15 min and held for 5 min (flow rate 0.30 mL/min, column temperature 50 °C, injection volume 1 µL). The MS scans were performed under the following conditions: gas temperature 250 °C, drying gas flow 8 L/min, nebulizer 35 psig, sheath gas temperature 375 °C, sheath gas flow 11 L/min, capillary voltage 1000 V, and fragmentor voltage 150 V. Mass spectra were recorded as centroid data for *m*/*z* 100–1000 in MS mode and *m*/*z* 40–1000 in MS/MS mode, with an acquisition rate of 14.0 spectra/sec. The automated MS/MS data-dependent acquisition was done for ions detected in the full scan above 2000 counts with a cycle time of 0.5 s, a quadrupole isolation width in narrow ~1.3 Da, using fixed collision energies of 10, 20, and 40 eV, and a maximum of three selected precursor ions per cycle. The instrument was tuned in low mass range (1700 *m*/*z*) and in extended dynamic range (2 GHz) mode. Under those conditions, the instrument was expected to provide experimental data with accuracy within ±3 ppm. Agilent MassHunter Data Acquisition and Qualitative Analysis Workflows (version B.08.00) software were used to acquire and process the data, respectively. The compound identification procedure is described in detail by Kramberger, et al. [13]. Semiquantitative comparison of different *H. italicum* samples was made by comparing abundances obtained from extracted ion chromatograms of individually identified compounds. Identified compounds were sorted by the chemical classes and subclasses and their abundances were summed.

### 2.5. Radical Scavenging Activity Assay

1,1-diphenyl-2-picrylhydrazyl (DPPH) reagent was dissolved in methanol, and samples of infusions were diluted in water. Ascorbic acid was used as a positive control. Samples were mixed with 0.2 mM DPPH methanol solution and incubated for 1 h at room temperature in the dark. To determine the reduction of DPPH, radical absorbance at 515 nm was measured on spectrophotometer Infinite F200 (Tecan Group Ltd., Zürich, Switzerland). The radical scavenging activity was calculated as a percentage of DPPH discoloration using the equation:

(A (sample with DPPH) − A (sample))/(A (DPPH) − A (solvent)) * 100,(1)

The presented results are for the 12.5% *v*/*v* concentration, which was used for all infusions in the linear range of the test. Each test was performed in five parallels and repeated twice.

### 2.6. Cell Cultures

Caco-2 human colorectal adenocarcinoma cells (ATCC^®^ HTB37™) and primary colon fibroblasts CCD112CoN (ATCC^®^ CRL1541™) were purchased from American Type Culture Collection (ATCC, Manassas, VA, USA) and cultured in Dulbecco’s Modified Eagle’s high glucose medium (DMEM) supplemented with 20% fetal bovine serum (FBS). U937 histiocytic lymphoma cells were purchased from ATCC (ATCC^®^ CRL-1593.2™) and grown in Roswell Park Memorial Institute (RPMI) culture media supplemented with 10% FBS. All cultures were maintained at 37 °C in a humidified atmosphere containing 5% CO_2_.

For all cell culture experiments, infusions were mixed directly with cell culture media, and the concentrations are expressed in *v*/*v* concentrations.

### 2.7. Cell ViabilityAassay

Cell viability after exposure to *Helichrysum* infusions was determined using PrestoBlue™ reagent (Invitrogen™, Carlsbad, CA, USA) according to the manufacturer’s instructions. Cells were treated with infusions diluted in cell culture media for 24 h. PrestoBlue™ was added and after 30 min, fluorescence was measured on a spectrophotometer Infinite F200 (Tecan Group Ltd., Zürich, Switzerland) at excitation/emission (ex/em) of 535/595 nm.

### 2.8. Intracellular ROS Level

Intracellular oxidative stress levels were evaluated using the 2ʹ,7ʹ-Dichlorofluorescin Diacetate (DCF-DA) assay [17]. Caco-2 and CCD112CoN cells were plated on black, clear-bottom 96-well plates at concentrations of 10.000 and 5.000 cells per well, respectively, and left to adhere to the plates for 24 h. U937 suspension cells were plated at a concentration of 10.000 cells per well. Cells were pretreated with different noncytotoxic dilutions of infusions for 24 h, whereas untreated samples served as controls. Cells were then washed in phosphate-buffered saline (PBS) and exposed to DCFH-DA to a final concentration of 50 μM and freshly prepared tert-butyl hydroperoxide solution (t-BOOH, 250 μM in PBS). The resulting increase in fluorescence was determined immediately and after 30 min incubation at room temperature using a spectrophotometer, Infinite F200 (Tecan Group Ltd., Zürich, Switzerland), at ex/em of 485/535 nm. The fluorescence increase is expressed as relative fluorescence, where the fluorescence of the control sample is set to 1.

### 2.9. Gene Expression Analysis

RNA was isolated from infusion-treated and control cells using TRIzol™ reagent (Thermo Fisher Scientific, Waltham, MA, USA) following the manufacturer’s instructions. One μg of RNA was reverse transcribed to cDNA with a cDNA Archive kit (Applied Biosystems, Foster City, CA, USA), and for the quantitative RT-PCR reactions, QuantStudio^®^ 3 Real-Time PCR System (Thermo Fisher Scientific, Waltham, MA, USA), SYBR Green master mix and 40 ng of cDNA template were used. The primer sequences 48762945c1 for superoxide dismutase 1 (SD1), 260436906c3 for catalase (CAT), and 305410788c1 for glutathione reductase (GR) were selected from PrimerBank [18] and used in a 0.5 µM final concentration. 18S rRNA was used as an internal control. Reaction conditions were 50 °C for 2 min, 95 °C for 10 min, and 40 cycles of 95 °C for 15 s and 60 °C for 1 min. Melting curves were inspected to ensure primer specificity. The results were analyzed using the ΔΔCt algorithm and are presented as the fold-change compared to non-treated cells.

### 2.10. Statistical Analysis

Values are presented as the mean ± standard deviation and were analyzed using statistical software (SPSS 23.0; IBM, Tokyo, Japan). Differences between groups were evaluated with one-way analysis of variance (ANOVA) followed by Dunnett’s test for multiple comparisons. Statistical significance was defined as *p* < 0.05.

## 3. Results

### 3.1. Plant Identification and Characterization

**Figure 1:**
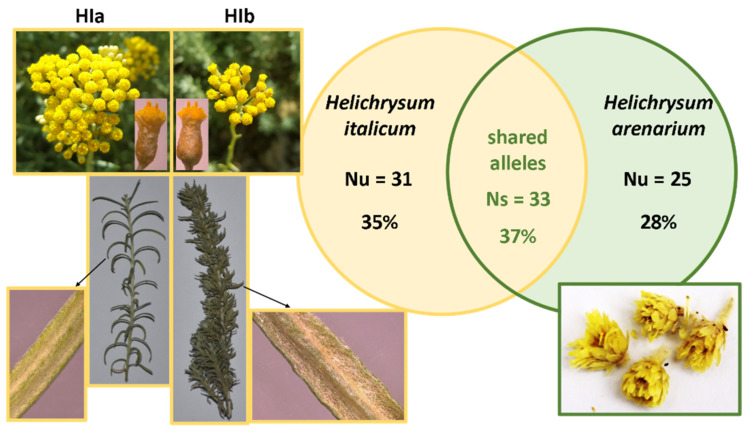
Plant material characterization. Taxonomically relevant differences among two identified HI morphological types HIa and HIb, the number (Nu) and the proportion of taxon-specific (unique) alleles for HI (Nu = 31; 35%) and HA (Nu = 25; 28%), and the number (Ns) and proportion of shared alleles among HI and HA (Ns = 33; 37%).

In the literature, there is often not enough information about the plant material used in the study. It is well known that both environmental factors and genotype can influence the chemical composition of a plant and consequently its biological effects. Therefore, we used genetic analysis to characterize both the commercially available product HA and the two morphological types of HI (HIa and HIb) grown in the commercial plantation and experimental collection, respectively. Newly developed microsatellite markers for HI [15] were successfully amplified in all analyzed plant materials. Examination of the eight microsatellite markers revealed 64 alleles in HI (HIa and HIb) and 58 alleles in HA. The number of amplified alleles identified in both species was similar, which indicates comparable polymorphism detected in the two species. A total of 89 alleles were detected, with the number of alleles per locus ranging from eight for loci HiUP-13 and HiUP-16 to 17 for locus HiUP-18. Microsatellite analysis identified a large number of alleles specific to each of the *Helichrysum* species. Eight microsatellite loci exhibited 31 (35%) HI specific (unique) alleles (Nu) and 25 (28%) HA unique alleles (Nu). The alleles of seven microsatellite markers (HiUP-02, HiUP-09, HiUP-13, HiUP-16, HiUP-18, HiUP-22, HiUP-24) shared by HI and HA (Ns) were 33 (37%). Only at locus HiUP-01 were no shared alleles found, and microsatellite profiles were unique for each of the species. In addition, microsatellite analysis revealed no substantial genetic differences between HIa and HIb genotypes, despite clear morphological differences between these two morphological types. Morphological differences between HIa and HIb, the total number of amplified alleles for HI and HA, and the proportion of shared alleles between HI and HA are presented in Figure 1.

The main chemical compounds which could be responsible for the biological effects, such as antioxidative potential and induction of gene expression, were analyzed (Table 1). Chemical composition comparison was done based only on the targeted compounds identified in our previous study [13]. The analysis is semiquantitative and therefore the relative abundances were used for the comparison between infusions [19]. The sum of the identified phenolic compounds was highest in HIa, whereas in HIb it was two-fold lower, and the lowest in HA. Looking at separate classes and subclasses of the identified compounds, we found hydroxycinnamic acids and total arzanol derivatives and other pyrones to be the most abundant in HIa. The representatives of hydroxycinnamic acids which were particularly abundant were caffeoylquinic and dicaffeoylquinic acids, while caffeic acid and its derivatives were more abundant in HIb. HIa was also the richest in cyclic polyols, isobenzofuranones, and neolignans. Only in this infusion were kaempferol and quercetin derivatives detected, and there were no flavanones. Subclasses of monohydroxybenzoic acids and coumarins were present in the largest quantities in HIb, and unlike the other two infusions, HIb also contained acetophenones and tremetones. The HA infusion was the richest in total flavonoids, especially flavanones. The abundances of caffeoylquinic acid, total hydroxybenzoic acids, and cyclic polyols in HA were not substantially lower than those in HIa. On the other hand, some flavonoid subclasses such as flavonols and flavones in HA were not detected. Furthermore, a difference from HI was observed in the absence of other hydroxycinnamic acids, pyrones, and coumarins.

### 3.2. Radical Scavenging Activity

**Figure 2:**
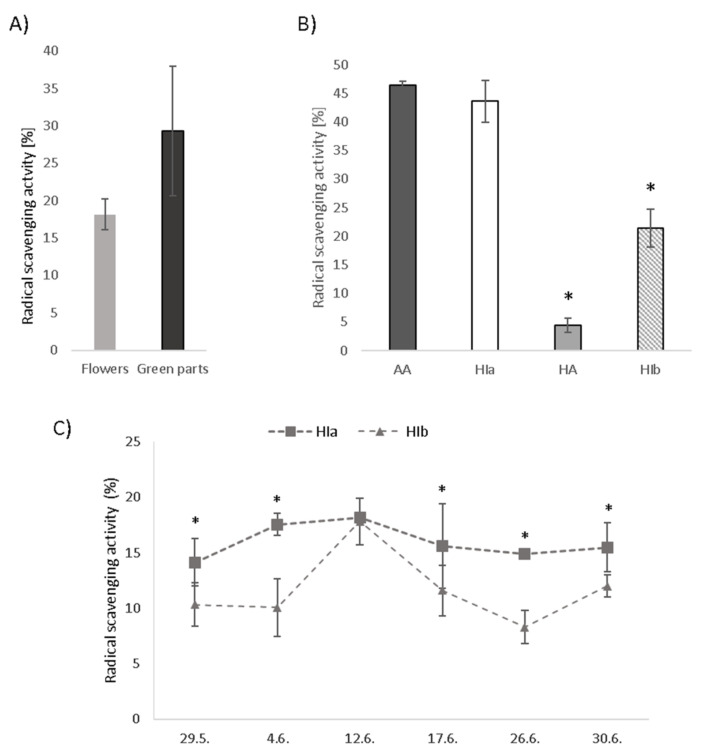
Radical scavenging activity measured by the DPPH test in (**A**) infusions prepared from flowers and green parts of HIa, (**B**) HIa, HIb, and HA infusions in comparison to 25 µM ascorbic acid (AA) and (**C**) HIa and HIb collected on different dates, all at a 12.5% *v*/*v* concentration. Mean ± SD is presented; * *p* < 0.05.

The DPPH test was used to determine the in vitro antioxidative potential of infusions. Green parts of HI exhibited higher radical scavenging activity compared to flowers alone (Figure 2A). Due to this reason and regardless of the fact that commercially available product contained only flowers, we decided to use whole aerial parts of HIa and HIb to fully exploit their putative health benefits. A comparison of infusions prepared from different species and morphological types revealed the highest radical scavenging activity of HIa, which was in the tested concentration of 12.5% *v*/*v* comparable to the activity of 25 µM ascorbic acid (AA) and reached 43.6% inhibition of DPPH (Figure 2B). The infusion of HIb at the same concentration inhibited 21.4% of DPPH, and the infusion of HA inhibited only 4.4%. As it was observed that the two HI types on a particular date were not always at the same growth stage and their scent was variably strong, in 2020, plant material was collected from both types on several dates during May and June. DPPH revealed only mild variability of radical scavenging activity for HIa. On the contrary, for HIb, the activity markedly varied and was on all tested dates significantly lower than that of HIa, with the exception of June the 12th, when the activities were comparable (Figure 2C).

### 3.3. Cytotoxicity and the Protective Effect from Oxidative Stress Induction

**Figure 3:**
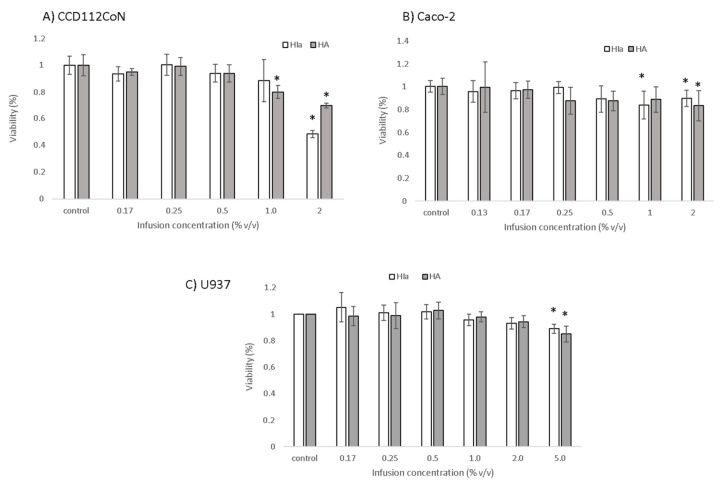
Cytotoxicity of HIa and HA infusions as determined by the PrestoBlue Assay in (**A**) CCD112CoN cells, (**B**) Caco-2 cells, and (**C**) U937 cells. The mean ± SD of three separate experiments is presented; * *p* < 0.05.

Due to higher radical scavenging activity, HIa was selected for the analysis in cell models in order to compare its effects to commercially available HA. The cytotoxicity of both infusions was inspected for cancerous colon cells Caco-2, primary colon cells CCD112CoN, and the lymphoma U937 cell line with PrestoBlue™ assay, and the results are presented in Figure 3. Infusions prepared from HIa and HA were for U937 cells toxic at 5% *v*/*v* concentration, whereas for Caco-2 and CCD112CoN, cells of one of the two were toxic already at a 1% *v*/*v* concentration. The HIa infusion at 1% *v*/*v* concentration was toxic for Caco-2 cells but not for the primary colon cells. For further experiments, non-cytotoxic test concentrations were used.

**Figure 4:**
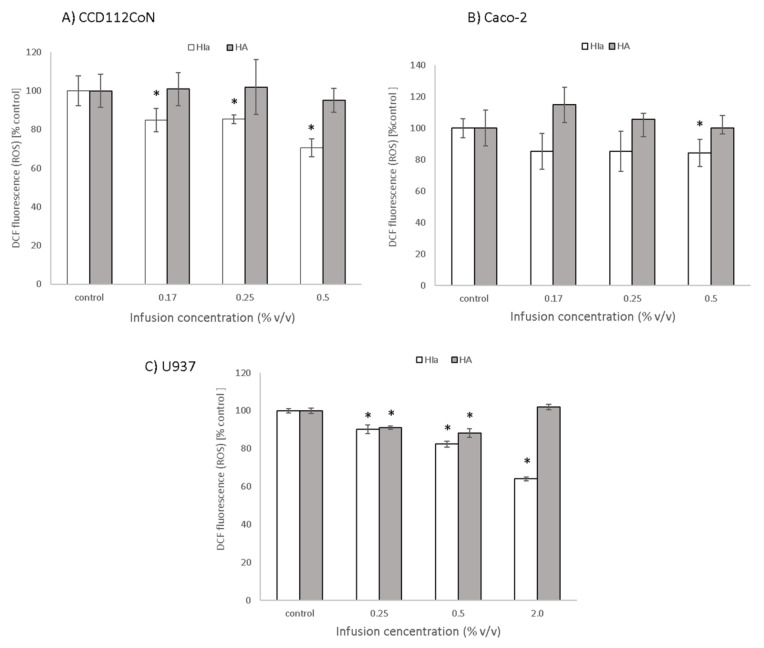
Protective effect of infusions against oxidative stress induction, determined with DCFH-DA assay in (**A**) CCD112CoN, (**B**) Caco-2, and (**C**) U937 cells. The mean ± SD of two separate experiments is presented; * *p* < 0.05.

To determine the protective effect of infusions against oxidative stress induction, DCFH-DA assay was employed. In Caco-2 cells, HIa at the highest tested concentration (0.5% *v*/*v*) reduced the level of reactive oxygen species concentrations to 84.2 ± 8%. The effect of HIa was even more pronounced in primary colon CCD112CoN cells, where it increased with concentration and reached a reduction of 70.5 ± 4.5% at a 0.5% (*v*/*v*) concentration of HIa infusion. HA had no significant effect on cancerous or on primary colon cells (Figure 4A,B). HIa was also more efficient than HA in reducing the levels of ROS in U937 cells; at the highest tested concentration of 2% (*v*/*v*), it reduced ROS to 64.1 ± 1%. HA at lower concentrations reduced the ROS level to 88.3 ± 1.6%, but the effect disappeared when a higher infusion concentration was used (Figure 4C).

### 3.4. The Expression of Genes Related to Oxidative Stress

**Figure 5:**
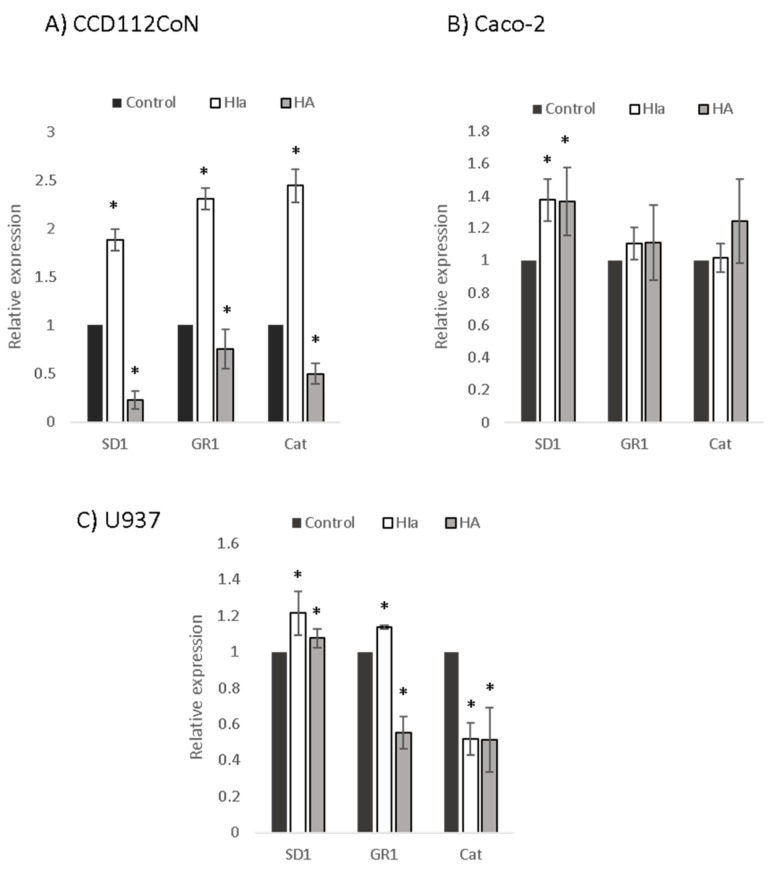
Expression levels of antioxidative enzymes. RT–PCR was performed to analyze the expressions of superoxide dismutase 1 (SD1), glutathione reductase 1 (GR1), and catalase (Cat) in (**A**) CD112CoN, (**B**) Caco-2, and (**C**) U937 cells. 18S rRNA was used as an endogenous control; the relative gene expression of non-treated cells was set to one, * *p* < 0.05.

To explore the mechanism of the antioxidative activity of *Helichrysum* infusions, the expression of three genes related to oxidative stress (SD1, GR1, and CAT) was determined by RT–PCR in infusion-treated cells. Similar to the determined protective effects, the greatest differences between the two infusions were detected in the primary colon cells (Figure 5). Here, HIa caused a significant 2–2.5-fold upregulation of all three enzymes; HA, on the contrary, downregulated all three genes. In Caco-2 cells, both infusions comparably upregulated the expression of SD1 (1.4-fold). In U937 cells, the HIa infusion caused a slight but significant increase in the expression of SD1 and GR1 and a decrease in the expression of CAT, whereas HA decreased the expression of both GR1 and CAT (Figure 5).

## 4. Discussion

Genetic analysis with microsatellites confirmed a clear differentiation among HI and HA species. In both *Helichrysum* species, unique alleles, characteristic of a defined species were found, confirming the usefulness of eight HiUP microsatellites in the identification process and traceability of the plant material. In addition, HI and HA shared more than one third of all detected alleles, indicating a common genetic background. Despite remarkable differences in the morphology and chemical profiles of plants HIa and HIb, these two forms shared identical alleles. Nevertheless, HIb was characterized by unique allele combinations allowing clear differentiation among the two morphs. The plant HIb is probably a hybrid, generated through spontaneous hybridization from unknown HI subspecies, which usually occur in the nature, where different subspecies of HI overlap [14,20]. Differences in the chemical composition, genetic structure, and antioxidative effects of *Helichrysum* species confirmed that accurate authentication is very important to ensure the most suitable and safe use of medicinal plants.

Infusions prepared from HI exhibited superior antioxidative properties to the infusion from a commercially available and approved medicinal plant of HA. This was true for both morphologically distinct HI plants used here; in vitro tests revealed the highest radical scavenging activity for HIa, followed by HIb, and HA. Furthermore, HIa was able to protect primary colon cells, colon cancerous cells, and lymphocytes from induced oxidative stress in a dose-dependent manner. HA also exerted a protective effect, but only in lymphocytes, and not at all tested concentrations. It can be pointed out that HIa was toxic to tumor Caco-2 cells at a lower concentration than in primary colon cells, which is an additional benefit, but for any claims about antitumorigenic effects, further studies should be performed.

The observations discussed above can be attributed to the chemical composition of the tested infusions. The total content of all identified phenolic compounds was substantial in all tested infusions, but there were considerable differences in the amounts of individual compounds. The content of total arzanol derivatives and other pyrones in the HIa infusion was approximately 50-fold higher than that in the HA infusion. Arzanol is one of the best investigated compounds found in HI and was previously found to exert strong antioxidative effects such as inhibition of lipid peroxidation [21]. The content of total hydroxycinnamic acids, for which the antioxidative potential was confirmed for many representatives, such as free caffeic acid [22], caffeoylquinic acids, and dicaffeoylquinic acids [23], was also the highest in HIa. The same was true for total flavonols, of which kaempferol derivatives, most likely also responsible for the somewhat more bitter taste, were the most abundant. HIa was the only infusion containing quercetin and its derivatives, which have a strong antioxidant activity for use in inflammatory conditions, mood disorders, and circulatory disfunction [24]. The total amount of flavonoids was, on the contrary, higher in HA, mostly due to the high content of flavanones. The latter was also expected, as apigenin, naringenin, and their derivatives were previously identified as major antioxidative constituents of HA methanol extract and tea made from inflorescences [25,26]. Previous studies on organic solvent-extracts of HA reported the presence of hydroxycinnamic, hydroxybenzoic acids, kaempferol, and low levels of quercetin [25,27]. While we detected representatives of the first two classes in the infusion of HA, kaempferol and quercetin were not detected. A chemical profile of HI infusions was also similar to that in previous reports; previously identified major polar constituents in methanol extract of HI flowers were caffeic acid, chlorogenic acid, quercetin derivatives, caffeoylquinic and dicaffeoylquinic acids, and tiliroside [28]. In line with our results, pyrone derivatives, such as mycropyrone, arzanol, and its derivatives were also reported [29]. On the contrary, flavanones such as naringenin were not detected in the HIa infusion, but were previously found in the methanolic extracts of the same species [30]. It is, however, important to stress that most previous chemical composition analyses were focused on organic solvent extracts, and the extraction technique largely influences the chemical composition [13,30].

Commercially available tea of HA contains only dried flowers, as only these are recognized as a herbal drug by the HMPC. We show here that for HIa, the antioxidative properties of green parts (stems and leaves) are superior to those of flowers, which led to a decision to use all aerial parts for the cell-culture experiments. Use of all aerial parts is also reported in ethnomedicine; fumes from flowers and leaves were reported to help with sleeplessness and headache and in decoctions against stomach-ache [31]. Infusions made from all aerial parts were used against cold [32] and to treat allergies [33]. Since green parts of HA were not available, we cannot exclude that these parts may also contain bioactive compounds which could contribute to the higher antioxidative potential of HA.

Based on the chemical analysis, it can be concluded that recreational tea prepared from HI or HA can be an important non-caloric source of health beneficial phenolic compounds. Despite the fact that some of these compounds are widely available in the diet, the intake of flavonols, for example, ranges only from 9 to 36.2 mg/day in the USA [34] and is comparatively low in Sweden and Norway [35]. The absence of caffeine, for those who wish to avoid its intake, makes *Helichrysum* infusions a good alternative to some other popular antioxidant-rich beverages, such as green tea.

To investigate whether the differential protective effects were exclusively due to the antioxidative properties of the infusions alone or also due to the differential response of particular cells to the treatments, the expressions of antioxidative enzymes were analyzed. It was shown for *Helichrysum plicatum* DC. that the extracts can increase the activities of antioxidative enzymes SD, GPx, and CAT when the concentration is not too high [36]. The effect on the expression of genes relevant in the response against oxidative stress may partly explain the mechanism through which HI reduces induced oxidative stress. In primary colon cells, where the protective effect was the most efficient, HIa infusion caused the most significant upregulation of SD1. In lymphocytes, both SD1 and GR1 upregulation in HIa-treated cells was consistent with the observed protective effect. In Caco-2 cells, no difference in the expression levels of any tested genes was observed, pointing to other possible mechanisms. A higher potential of the infusion itself is one possible explanation, whereas changes in the expression of other genes or in the production of endogenous antioxidants are also possible.

The principle of hormesis, a well-known phenomenon, where a mild stress caused by a low dose of a particular compound induces adaptive responses in cells, preparing them for forthcoming stressful events [37,38], could also partially explain our observations. Such a principle was previosly confirmed for several stressors, such as oxygen, nitric oxide, carbon monoxide, and physical exercise [37]. Among well-investigated stressors are plant extracts, such as extracts of Ginkgo biloba [39] and cruciferous vegetables [40], and/or single phenolic compounds [41,42]. In the present study, the protective effect against induced stress was indeed observed in cells pretreated with the infusions and the upregulation of the genes, coding for the antioxidative enzymes, could be one of such adaptations. The main idea of hormetic activity is that a stressor in a low dose induces a mild stress to which cells respond with beneficial overcompensation to the damaging stimulus, whereas at higher doses, the damaging effects predominate [38]. The fact that the HA infusion in U937 cells was protective at a lower but not a higher concentration may point to such a mechanism.

For the initial experiments, plant material was collected in early June 2019. In the following weeks, we noticed that the scent of the plants varied. Even though the scent depends on volatile compounds which are not responsible for the antioxidative properties of infusions, these differences could indicate that the content of bioactive compounds changes over time. The same has been observed for other plants [43]. This was confirmed when plant material was collected at different time points in 2020. While HIa exerted a stable antioxidative potential, HIb had an obvious peak in mid-June. The result suggests that the harvesting time has an important influence on the quality of the harvested material. However, the optimal harvest period cannot be suggested based solely on our data, as it may depend not only on the species/subspecies but also on the specific weather conditions. Observing the antioxidative properties over a longer period of time in several seasons with special attention on the phenophase of the plant would provide the most useful and transferable information.

## 5. Conclusions

Taken together, our results provide scientific confirmation of the health beneficial value of traditionally used HI infusions and answer, at least in part, the frequently expressed call for such data in the literature. The superior antioxidative potential of HI compared to HA observed here may suggest that HI could also be evaluated as a medicinal plant, and further in vivo studies can be recommended. At the same time, we show that the two distinct morphological types are different in terms of chemical composition and consequent functional properties. This points to the importance of thorough plant characterization prior to plantation set-up. Additionally, the harvesting time also importantly contributes to the antioxidative effects of the infusions, which suggests this parameter is crucial and should be considered to obtain the best possible product and thus fully exploit its commercial potential.

## Figures and Tables

**Table 1:** HPLC–MS analysis of the infusions. Semiquantitative analysis was preformed to compare the abundances of the main components in HIa, Hib, and HA. The relative abundances are presented for each infusion. Calculations were performed based on abundance summation of individual compounds belonging to each subclass and class. Names of subclasses are written in italics.

Compound Name	Infusion		
	HIa	HIb	HA
**Total Identified Phenolic Compounds ^1^**	**2071068**	**974921**	**681606**
Total hydroxycinnamic acids	1104310	472357	189173
*Caffeic acid and its derivatives*	*174492*	*218381*	*31144*
*Other hydroxycinnamic acids and their derivatives*	*43618*	*44407*	*0*
*Caffeoylquinic acids*	*443858*	*197797*	*150534*
*Other monoesters*	*59450*	*0*	*4167*
*Dicaffeoylquinic acids*	*382892*	*11772*	*3328*
Total hydroxybenzoic acids	89445	92466	64752
*Monohydroxybenzoic acids*	*43026*	*72816*	*42956*
*Dihydroxybenzoic acids*	*46419*	*19650*	*21796*
Total flavonoids	92004	24180	227018
*Quercetin derivatives*	*25573*	*0*	*0*
*Myricetin derivatives*	*10056*	*5223*	*0*
*Kaempferol derivatives*	*47970*	*0*	*0*
*Flavanones*	*0*	*18957*	*227018*
*Flavones*	*8405*	*0*	*0*
Total arzanol derivatives and other pyrones	392448	275723	7521
*Pyrones*	*384837*	*256991*	*0*
*Arzanol and its derivatives*	*7611*	*18732*	*7521*
Total other phenolic compounds	392861	110195	193142
*Cyclic polyols*	*180368*	*54374*	*168071*
*Isobenzofuranones*	*156472*	*4638*	*22703*
*Neolignans*	*48243*	*15502*	*2368*
*Acetophenones*	*0*	*3750*	*0*
*Tremetones* *Coumarins*	*0* *7778*	*14525* *17406*	*0* *0*

^1^ Sum of total abundances of all compound classes (hydroxycinnamic acids, hydroxybenzoic acids, flavonoids, arzanol derivatives, other pyrones, and other phenolic compounds) listed in the table.

## Data Availability

The data presented in this study are available on request from the corresponding author.
